# Comparison of scores for the classification of cardiometabolic risk in adult patients enrolled in a Venezuelan program for chronic non-communicable diseases: a cross-sectional study

**DOI:** 10.1590/1516-3180.2019.0236.R1.06112019

**Published:** 2020-04-22

**Authors:** Nelina Alejandra Ruíz-Fernández, Ulises Leal, Milagros Espinoza

**Affiliations:** I PhD. Medical Laboratory Technician and Professor, Department of Morphophysiopathology, School of Bioanalysis, Faculty of Health Sciences, Universidad de Carabobo, Valencia, Carabobo, Venezuela; and Principal Researcher, Institute of Nutritional Research, Faculty of Health Sciences, Universidad de Carabobo, Valencia, Carabobo, Venezuela.; II MD. Physician and Internal Medicine Specialist, Integral Medical Care Unit, University of Carabobo, Valencia, Carabobo, Venezuela; and Specialist Physician type II, Outpatient Clinic of the Municipality of San Diego, Carabobo, Venezuela.; III PhD. Medical Laboratory Technician and Professor, Department of Research and Professional Development, School of Bioanalysis, Faculty of Health Sciences, Universidad de Carabobo, Valencia, Carabobo, Venezuela.

**Keywords:** Cardiovascular diseases, Diabetes mellitus, Metabolic syndrome, Risk factors, Cardiovascular risk, Cardiometabolic diseases, Cardiometabolic indexes, Syndrome X, Continuous risk scores, Clustered cardiovascular factors

## Abstract

**BACKGROUND:**

Several continuous measurements of cardiometabolic risk (CMR) have emerged as indexes or scores. To our knowledge, there are no published data on its application and validation in Latin America.

**OBJECTIVE:**

To evaluate four continuous measurements of metabolic status and CMR. We established its predictive capacity for four conditions associated with CMR.

**DESIGN AND SETTING:**

Cross-sectional study conducted at a healthcare center in the state of Carabobo, Venezuela.

**METHODS:**

The sample comprised 176 Venezuelan adults enrolled in a chronic disease care program. Four CMR scores were calculated: metabolic syndrome (MetS) Z-score; cardiometabolic index (ICMet); simple method for quantifying MetS (siMS) score; and siMS risk score. CMR biomarkers, proinflammatory status and glomerular function were assessed. MetS was established in accordance with a harmonized definition.

**RESULTS:**

Patients with MetS showed higher levels of all scores. All scores increased as the number of MetS components rose. The scores showed significant correlations with most CMR biomarkers, inflammation and glomerular function after adjusting for age and sex. In the entire sample, MetS Z-score, siMS score and siMS risk score showed the ability to detect MetS, reduced glycemic control, proinflammatory status and decreased estimated glomerular filtration rate. ICMet only discriminated MetS and proinflammatory state. There were some differences in the predictive capacity of the scores according to sex.

**CONCLUSIONS:**

The findings support the use of the scores assessed here. Follow-up studies should evaluate the predictive capacity of scores for cardiovascular events and diabetes in the Venezuelan population.

## INTRODUCTION

Cardiometabolic diseases are responsible for a significant number of deaths around the world.^1^ In the year 2013, heart disease, diabetes mellitus and cerebrovascular diseases ranked as first, third and fourth leading causes of mortality in Venezuela.^2^

The likelihood of developing cardiovascular disease and diabetes mellitus is known as cardiometabolic risk. The etiopathogenesis of these diseases is complex and involves a wide range of interconnected cardiometabolic risk factors that often match in the same patient. The term metabolic syndrome describes the confluence of cardiometabolic risk factors in an individual, such as abdominal obesity, atherogenic dyslipidemia, hypertension, glucose intolerance/insulin resistance, microalbuminuria, proinflammatory and prothrombotic state. Presence of metabolic syndrome raises the cardiometabolic risk because this syndrome confers a fivefold increase in the risk of diabetes mellitus type 2 and a twofold increase in the risk of cardiovascular disease over the next five to ten years.^3^

There are various diagnostic criteria for metabolic syndrome, but in all cases, the diagnosis ends up using a cutoff point for a dichotomous response: absence or presence of metabolic syndrome when at least three of the five individual components of metabolic syndrome are accumulated. However, the expression of each risk component is continuous, and cardiometabolic risk is a progressive function of these combined risk measurements.^4^^,^^5^^,^^6^ Use of dichotomous definitions for metabolic syndrome denies the scientific evidence, since it is a gradual condition in which the risk spectrum increases progressively with the number of anomalies or individual factors accumulated.^7^^,^^8^ Also, the conventional diagnosis of metabolic syndrome does not make it possible to follow up the gradual changes that occur in individuals with metabolic syndrome once the therapeutic measures are in place.^8^

A continuous cardiometabolic risk index responds to the above limitations. It shows the continuous risk to which an individual is exposed and provides information about the severity of the risk. Over recent years, several continuous measurements of cardiometabolic risk have emerged as indexes or scores. In general, these include the same individual components of metabolic syndrome but differ in the methodologies that are used for their construction and calculation.

None of these proposed continuous scores for metabolic syndrome originated from the Latin American population. To our knowledge, there are no published data on application and validation of such scores in Latin America. It is important to consider that the prevalence of this disease, its survival rates and the distribution of risk factors and their weights as determinants of the disease may be different in each population.^9^ There is also genetic and environmental control over the expression of cardiometabolic risk factors in each population group.

## OBJECTIVE

The aim of this research was to evaluate four continuous measurements of metabolic status and cardiometabolic risk in a group of adult patients who had been enrolled in the CAREMT (Cardio Renal Endocrine Metabolic and Tobacco) program, which was developed at a healthcare center in the state of Carabobo, Venezuela. We explored the variation of continuous measurements according to different biomarkers for cardiometabolic risk, inflammation and glomerular function. We established the ability of continuous measurements to discriminate or detect metabolic syndrome, reduced metabolic control, proinflammatory status and decreased glomerular function. This exploratory assessment was the first step towards validation of continuous measurements of cardiometabolic risk in Latin American countries such as Venezuela, for future primary care applications.

## METHODS

### Participants and data collection procedure

This was a cross-sectional study of correlational type, with a non-experimental design. The validation of continuous measurements was performed using a cross-section of baseline data from the CAREMT program, implemented at a primary healthcare center in the state of Carabobo, Venezuela. This program consists of an integration of the cardiovascular, endocrine, metabolic, renal, cancer and anti-smoking programs, in a strategy for screening and prevention of the most frequent non-communicable chronic diseases and their risk factors.

The Research Ethics Committee of the University of Carabobo approved this study (CPBBUC-002-2019-DIC-NR; code: KE94KD90; date: May 9, 2019). The study procedures followed the ethical standards of the Helsinki Declaration and its revisions. Informed consent was obtained from each participant.

The study was based on non-probabilistic sampling. The population comprised all the adult patients (20-65 years of age) of both genders who were enrolled in the CAREMT program between 2015 and August 2017 (n = 210). The sample was composed of 176 patients, after exclusion of patients with one or more of the following conditions: personal antecedents of cardiovascular or cerebrovascular events; body mass index under 18.5 kg/m² or greater than 35 kg/m²; significant alterations in muscle mass (amputations, loss of muscle mass, muscle diseases or paralysis); renal failure; pregnancy; lactation; severe hepatopathy; generalized edema; ascites; or incomplete anthropometric measurements or biochemical determinations.

We applied an instrument for collecting personal and biomedical data. The same interviewer always performed the interview to ensure standardization of the procedure. The participants underwent anthropometric-clinical measurements and a blood sample was taken. They were instructed to have a light dinner and to fast for 12 hours before blood collection. A partial morning urine sample was requested on the day when blood was collected.

### Anthropometric, blood pressure and biochemical measurements

Weight and height measurements were made following standard protocols. Waist circumference was measured with a measuring tape at the midpoint between the last rib and the iliac crest, with the subject standing. This measurement was made at the end of an unstressed expiration. The waist circumference, body mass index (BMI; in kg/m^2^) and waist-to-height ratio (WHR; in cm/cm) were classified as elevated in accordance with the accepted criteria.^10^^,^^11^^,^^12^

Blood pressure was measured using a sphygmomanometer (Omron model M7; Omron Health Care, Kyoto, Japan). The diagnosis of arterial hypertension was established in accordance with international recommendations.^13^ The percentage of body fat (%BF) was ascertained using a body composition analyzer (model TBF 300 A; Tanita, Tokyo, Japan). %BF ≥ 25% (men) and ≥ 30% (women) was considered elevated.^14^

The A1_C_ hemoglobin fraction (HbA1_C_) in whole blood was assessed by means of an immunoassay. Glucose, creatinine, triglycerides (TGL), total cholesterol (TC), low-density lipoprotein-cholesterol (LDLc) and high-density lipoprotein-cholesterol (HDLc) were determined in serum using colorimetric enzymatic methods. Serum high-sensitivity C*-*reactive protein (hsCRP) was quantified by means of immunoturbidimetry. The protein content in the partial urine sample was determined using a reactive tape. Detection of a protein level of at least one cross (+) in the urine was defined as proteinuria.

TC/HDLc, LDLc/HDLc and TGL/HDLc ratios and the non-HDL cholesterol concentration (TC-HDLc) were calculated. The estimated glomerular filtration rate (eGFR) was obtained through the Chronic Kidney Disease Epidemiology Collaboration (CKD-EPI) equation,^15^ using the renal function calculator of the Spanish Society of Nephrology.^16^

Presence of metabolic syndrome and its individual components were established in accordance with a harmonized definition.^11^ Presence of diabetes was defined using the criterion of the American Diabetes Association.^17^ TC, LDLc, TC/HDLc ratio, LDLc/HDLc ratio and non-HDL cholesterol were classified as elevated in accordance with previously described criteria.^18^^,^^19^^,^^20^^,^^21^

Existence of a proinflammatory state was defined as a hsCRP level ≥ 1 mg/l. In addition, the hsCRP level was classified as indicative of average cardiovascular risk when it was 1-3 mg/l or as indicative of high cardiovascular risk when it was ≥ 3.0 mg/l.^22^ The level of glycemic control was categorized as “reduced” when HbA1_C_ was ≥ 5.7%; additionally, HbA1_C_ was categorized as < 5.7% (normal), 5.7%-6.4% (prediabetes) or ≥ 6.5% (diabetes).^17^ eGFR was defined decreased using the cutoff points recommended through the guidelines of the National Kidney Foundation.^23^

### Continuous scores for cardiometabolic risk

The following continuous scores for cardiometabolic risk were evaluated:

- *Continuous metabolic syndrome severity Z-score (MetS Z-score)*: this was calculated by applying the equations proposed by Gurka et al.^24^ for Hispanic individuals according to sex, using the calculator available at http://mets.health-outcomes-policy.ufl.edu/calculator/.
- *Cardiometabolic index (ICMet)*: Wakabayashi and Daimon^25^ proposed this index. It was calculated as the product of the TGL/HDLc ratio and WHR.
- *Simple method for quantifying metabolic syndrome (siMS) score and siMS risk score)*: Soldatovic et al.^26^ proposed these continuous scores. The first assesses the state of metabolic syndrome and the second evaluates the risk of coronary heart disease or cerebrovascular events. These scores were determined using the spreadsheet provided by Soldatovic et al.^26^ and introducing the cutoff points of the metabolic syndrome definition applied in the present study.

### Statistical analysis

Statistical Package for the Social Sciences (SPSS) software, version 20.0.0 for Windows (SPSS, Chicago, IL, USA), except for the receiver operating characteristic curves (ROC curves) and their parameters. ROC curves were obtained through the MedCalc software, in its version 13.3.3.0 for Windows.

The variables studied were assessed with regard to normality of distribution, by means of the Kolmogorov-Smirnov test. Variables that did not follow this distribution were transformed using the process described by Templeton.^27^

Means, standard deviations, medians, interquartile ranges and absolute and relative frequencies were used to characterize the sample. To correlate the frequency of cardiovascular risk factors with sex, the chi-square test was applied. The unpaired Student t test or the Mann-Whitney U test was used, as appropriate, to compare the variables according to sex, age groups and metabolic syndrome. The age groups were formed according to the median age for each sex. The Kruskal-Wallis test or Mann-Whitney U test was used, as appropriate, to compare scores according to the numbers of individual metabolic syndrome components and categories of biomarkers for cardiometabolic risk, inflammation and glomerular function.

Multiple linear regression analysis was conducted to assess the relationship between the continuous scores and the different biomarkers, with adjustment for age and sex (the back method was used for introducing variables). ROC curves for the continuous scores were constructed to test their predictive value for detecting metabolic syndrome, reduced glycemic control, proinflammatory state and decreased estimated glomerular function. The area under the curve (AUC) and its 95% confidence interval were obtained through a nonparametric method. The Hanley and McNeil method was used to compare the AUCs.

## RESULTS

Table 1 shows the characteristics of the sample according to age and sex. Women showed higher age and %BF, while men had higher values for weight, height, creatinine and eGFR. Among all the subjects, 36.9% had family antecedents of cardiovascular diseases, 5.7% reported being a smoker, 29.5% were diabetic, 43.8% were hypertensive, 42.0% were undergoing hypotensive treatment at the time of the evaluation, 62.5% had metabolic syndrome, 50.6 % had reduced glycemic control, 60.6% showed a proinflammatory state and 65.3% presented decreased eGFR. Diabetes, metabolic syndrome and decreased glomerular function were more frequent among men (P < 0.05).

**Table 1.** Characteristics of the study participants according to gender

Variables	Age group		**Entire sample**
Women	≤ 54 years (n = 53)	> 54 years (n = 41)	(n = 94)
Age	48.5 (44.2-52.8)^**^	58.0 (55.0-59.0)	54.0 (48.0-57.0)^‡^
Weight (kg)	67.0 (61.0-73.0)	65.0 (56.0-73.5)	66.0 (59.0-73.0)
Height (m^2^)	1.56 (1.52-1.61)	1.55 (1.48-1.60)	1.56 (1.50-1.60)
BMI (kg/m^2^)	27.5 (25.1-28.8)	27.3 (24.4-30.0)	27.5 (24.6-29.0)
WC (cm)	89.0 (84.5-92.0)	88.0 (81.5-94.0)	89.0 (82.8-92.2)
WHR	0.57 (0.53-0.59)	0.56 (0.52-0.61)	0.56 (0.51-0.61)
Body fat (%)	38.1 (33.4-41.0)	38.6 (36.8-44.4)	38.5 (35.0-41.8)^‡‡^
SBP (mmHg)	120.0 (102.0-139.5)	120.0 (120.0-140.0)	120.0 (110.0-140.0)
DBP (mmHg)	77.0 (70.0-83.8)	79.0 (70.0-80.0)	77.0 (70.0-80.0)
Glucose (mg/dl)	95.0 (88.2-112.5)	101.0 (89.5-113.0)	98.0 (88.8-111.8)
HbA1_C_ (%)	5.2 (4.4-6.2)	5.9 (4.9-6.9)	5.5 (4.5-6.5)
Creatinine (mg/dl)	0.9 (0.8-1.1)	0.9 (0.8-1.0)	0.9 (0.8-1.0)
TC (mg/dl)	215.5 (189.0-251.8)^**^	182.0 (152.5-234.5)	202.0 (174.5-239.5)
LDLc (mg/dl)	129.8 (110.8-156.4)^*^	107.4 (82.0-148.2)	123.1 (94.2-154.2)
HDLc (mg/dl)	43.0 (37.2-54.0)	43.0 (38.0-46.5)	43.0 (38.0-50.2)
TGL (mg/dl)	187.5 (121.5-254.0)	147.0 (107.0-195.0)	163.0 (114.0-220.0)
TC/HDLc ratio	5.2 (4.1-6.1)	4.5 (3.7-5.7)	4.8 (3.7-5.9)
LDLc/HDLc ratio	3.0 (2.3-4.0)	2.7 (1.9-3.8)	3.0 (2.0-3.9)
TGL/HDLc ratio	4.3 (2.4-6.1)	3.6 (2.7-4.5)	3.8 (2.7-5.3)
Non-HDL cholesterol (mg/dl)	171.0 (148.3-195.5)^**^	137.0 (109.0-188.0)	158.5 (132.0-192.5)
hsCRP (mg/l)	1.4 (0.7-3.0)	1.2 (0.9-3.4)	1.2 (0.8-3.0)
eGFR (ml/min/1.73 m^2^)	81.1 ± 18.4	75.6 ± 18.5	79 ± 18.6
siMS score	3.42 (3.00-3.92)	3.37 (3.01-3.63)	3.38 (3.01-3.86)
siMS risk score	3.69 (2.73-4.19)^**^	4.12 (3.53-4.85)	3.93 (3.24-4.54)
ICMet	2.30 (1.39-3.38)	2.08 (1.53-2.62)	2.14 (1.48-3.04)
MetS Z-score	0.61 (0.18-1.08)	0.61 (0.03-1.00)	0.61 (0.13-1.01)
Variables	Age group		Entire sample
Men	≤ 51 years (n = 46)	> 51 years (n = 36)	(n = 82)
Age	47.0 (44.0-50.0)^**^	55.5 (53.2-58.0)	51.0 (45.8-55.0)
Weight (kg)	74.0 (69.0-84.0)	70.3 (59.0-73.0)	72.5 (66.8-80.2)^‡‡^
Height (m^2^)	1.64 (1.56-1.70)	1.64 (1.55-1.70)	1.64 (1.56-1.70)^‡‡^
BMI (kg/m^2^)	28.2 (25.1-30.8)	26.8 (25.0-28.6)	27.2 (25.1-30.0)
WC (cm)	94.0 (85.8-100.0)	92.0 (82.5-98.0)	92.5 (84.0-98.2)^‡^
WHR	0.57 (0.51-0.62)	0.56 (0.51-0.60)	0.57 (0.51-0.61)
Body fat (%)	35.3 (27.3-38.5)	34.2 (27.2-39.6)	35.0 (27.3-38.6)
SBP (mmHg)	120.0 (110.0-130.0)	120.0 (110.0-140.0)	120.0 (110.0-137.2)
DBP (mmHg)	70.0 (70.0-80.0)	70.0 (70.0-80.0)	70.0 (70.0-80.0)
Glucose (mg/dl)	109.5 (93.5-161.0)	96.0 (84.8-120.0)	101.0 (89.0-140.8)
HbA1_C_ (%)	6.3 (4.5-7.8)^*^	5.4 (4.2-6.4)	5.9 (4.3-7.3)
Creatinine (mg/dl)	1.1 (0.9-1.1)	1.0 (0.8-1.1)	1.0 (0.9-1.1)^‡‡^
TC (mg/dl)	197.0 (166.7-233.0)	196.0 (167.0-229.0)	197.0 (166.8-230.5)
LDLc (mg/dl)	122.4 (86.6-145.7)	126.4 (91.4-140.9)	125.3 (90.8-140.8)
HDLc (mg/dl)	40.0 (37.5-50.5)	45.0 (40.0-52.8)	43.0 (38.8-52.0)
TGL (mg/dl)	187.5 (130.7-224.8)	153.5 (95.8-196.5)	168.0 (116.8-204.0)
TC/HDLc ratio	4.5 (3.7-6.3)	4.1 (3.6-5.2)	4.4 (3.6-5.6)
LDLc/HDLc ratio	2.8 (2.0-4.5)	2.5 (2.1-3.5)	2.7 (2.0-3.6)
TGL/HDLc ratio	4.4 (3.0-6.0)^**^	3.3 (2.0-4.3)	3.9 (2.4-5.2)
Non-HDL cholesterol (mg/dl)	161.0 (123.5-188.8)	150.5 (119.8-179.0)	152.0 (121.2-182.0)
hsCRP (mg/l)	1.7 (0.7-3.0)	1.0 (0.5-3.5)	1.2 (0.6-3.0)
eGFR (ml/min/1.73 m^2^)	88.7 ± 16.2	85.7 ± 15.9	87.4 ± 16.0^‡‡^
siMS score	3.40 (2.92-3.89)^*^	2.94 (2.59-3.49)	3.22 (2.71-3.66)
siMS risk score	3.64 (3.02-4.16)	3.99 (3.45-4.29)	3.75 (3.22-4.28)
ICMet	2.35 (1.77-3.19)^**^	1.82 (1.11-2.34)	2.14 (1.36-2.97)
MetS Z-score	0.66 (0.21-1.14)^*^	0.20 (-0.02-0.79)	0.46 (0.04-0.97)

Data expressed as mean ± standard deviation, median (interquartile range), n (%). Unpaired Student t test or Mann-Whitney U test, according to case. ^*^P < 0.05 and ^**^P < 0.01 between age groups. ^‡^P < 0.05 and ^‡‡^P < 0.01 between women and men.

BMI = body mass index; WC = waist circumference; WHR = waist to height ratio; SBP = systolic blood pressure; DBP = diastolic blood pressure; HbA1_C_ = A1_C_ hemoglobin fraction; TC = total cholesterol; LDLc = low-density lipoprotein cholesterol; HDLc = high-density lipoprotein cholesterol; TGL = triglycerides; hsCRP = ultrasensitive C-reactive protein; eGFR = estimated glomerular filtration rate.

**Figure 1.**
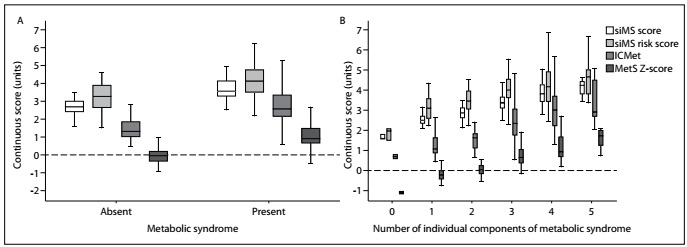
Continuous scores for cardiometabolic risk, evaluated according to A) presence of metabolic syndrome and B) number of metabolic components.

The medians for the siMS score, siMS risk score, ICMet and MetS Z-score were higher in patients with metabolic syndrome (P < 0.001) (Figure 1A). The scores studied increased as the number of individual metabolic syndrome components also increased (P < 0.001) (Figure 1B).

The variation of the scores according to the categories of the biomarkers assessed is shown in Table 2. All the scores were significantly higher in patients with elevated BMI, waist circumference, WHR, %BF, glucose, HbA1_C,_ TGL, TGL/HDLc ratio and hsCRP; all the scores were higher among patients with low HDLc. The siMS score, siMS risk score and MetS Z-score increased significantly as eGFR decreased; these indicators were also higher in patients with proteinuria. All the scores were significantly higher among diabetic patients. Only the siMS risk score was significantly higher among smokers and patients with a family history of cardiovascular disease; none of the scores was higher in hypertensive patients.

**Table 2.** Continuous scores for cardiometabolic risk, assessed according to biomarkers for cardiometabolic risk, inflammation and glomerular function in adult patients

Biomarkers		siMS score	siMS risk score	ICMet	MetS Z-score
BMI (kg/m^2^)	< 25	2.77 (2.40-3.46)	3.66 (2.66-4.12)	1.71 (1.03-2.31)	0.07 (-0.34-0.53)
	≥ 25	3.39 (3.02-3.87)^**^	3.95 (3.26-4.55 )^*^	2.20 (1.67-3.16)^**^	0.66 (0.26-1.13)^**^
WC (cm)	< 90 or 80	2.99 (2.54-3.45)	3.68 (2.86-4.10)	1.81 (1.08-2.32)	0.15 (-0.24-0.57)
	≥ 90 or 80	3.42 (3.03-3.96)^**^	3.93 (3.28-4.60)^*^	2.22 (1.64-3.36)^**^	0.71 (0.25-1.26)^**^
WHR	< 0.5	2.92 (2.42-3.31)	3.58 (2.64-4.00)	1.69 (1.03-2.16)	0.05 (-0.52-0.38)
	≥ 0.5	3.38 (2.93-3.92)^**^	3.91 (3.27-4.52)^**^	2.20 (1.59-3.12)^**^	0.65 (0.19-1.13)^**^
Body fat (%)	< 25 or 30	2.48 (1.64-3.49)	3.26 (1.96-4.06)	1.08 (0.70-2.31)	-0.12 (-0.98-0.69)
	≥ 25 or 30	3.34 (2.92-3.85)^**^	3.87 (3.24-4.48)^*^	2.14 (1.53-3.04)^*^	0.61 (0.09-1.01)^*^
SBP (mmHg)	< 130	3.34 (2.71-3.86)	3.68 (3.03-4.26)	2.15 (1.36-3.24)	0.56 (0.13-1.10)
	≥ 130	3.32 (3.00-3.65)	4.04 (3.43-4.48)^*^	2.12 (1.54-2.58)	0.60 (0.22-0.90)
DBP (mmHg)	< 85	3.31 (2.77-3.70)	3.86 (3.23-4.41)	2.13 (1.36-2.95)	0.53 (0.05-0.99)
	≥ 85	3.45 (3.02-3.88)	3.90 (3.12-4.48)	2.30 (1.63-3.45)	0.79 (0.26-1.04)
Glucose (mg/dl)	< 100	3.05 (2.61-3.42)	3.54 (2.80-4.11)	1.89 (1.24-2.57)	0.24 (-0.20-0.62)
	≥ 100	3.58 (3.19-4.31)^**^	4.04 (3.48-4.95)^**^	2.37 (1.64-3.26)^**^	0.97 (0.57-1.88)^*^
HbA1_C_ (%)	< 5.7	3.18 (2.67-3.52)	3.75 (3.00-4.15)	2.01 (1.36-2.60)	0.34 (-0.10-0.65)
	5.7-6.4	3.37 (2.77-3.64)	3.69 (3.21-4.24)	1.95 (1.17-2.57)	0.64 (0.15-0.98)^*^
	≥ 6.5	3.55 (3.13-4.54)^**,‡‡^	4.12 (3.40-5.19)^**,‡^	2.58 (1.76-3.43)^*,‡^	1.03 (0.46-2.14)^**,‡‡^
TC (mg/dl)	< 200	3.16 (2.68-3.70)	3.67 (2.99-4.30)	1.82 (1.15-2.94)	0.48 (-0.01-0.97)
	> 200	3.44 (3.09-3.85)^*^	3.93 (3.36-4.57)^*^	2.34 (1.85-3.02)^**^	0.61 (0.26-1.04)
LDLc (mg/dl)	≤ 130	3.31 (2.73-3.86)	3.75 (3.06-4.47)	2.08 (1.33-3.15)	0.61 (0.07-0.98)
	> 130	3.34 (2.94-3.64)	3.92 (3.30-4.34)	2.15 (1.66-2.86)	0.56 (-0.02-1.00)
HDLc (mg/dl)	> 40 or 50	2.99 (2.54-3.33)	3.49 (2.97-4.15)	1.70 (1.08-2.18)	0.22 (-0.23-0.57)
	< 40 or 50	3.56 (3.22-4.14)^**^	3.99 (3.43-4.72)^**^	2.58 (1.92-3.48)^**^	0.85 (0.43-1.38)^**^
TGL (mg/dl)	< 150	2.75 (2.44-3.15)	3.40 (2.78-3.98)	1.31 (0.99-1.79)	0.07 (-0.32-0.47)
	≥ 150	3.55 (3.30-4.21)^**^	4.09 (3.54-4.73)^**^	2.66 (2.14-3.55)^**^	0.79 (0.46-1.38)^**^
TC/HDLc ratio	≤ 5.0 or ≤ 4.5	3.06 (2.61-3.40)	3.69 (3.00-4.29)	1.72 (1.09-2.24)	0.24 (-0.11-0.72)
	> 5.0 or > 4.5	3.58 (3.33-4.14)^**^	3.96 (3.40-4.54)	2.62 (2.11-3.54)^**^	0.88 (0.46-1.34)^**^
LDLc/HDLc ratio	≤ 3.5 or ≤ 3.0	3.17 (2.66-3.65)	3.75 (3.06-4.29)	1.86 (1.16-2.49)	0.38 (-0.03-0.92)
	> 3.5 or > 3.0	3.46 (3.12-4.16)^**^	3.98 (3.38-4.57)	2.57 (1.90-3.39)^**^	0.79 (0.38-1.51)^**^
TGL/HDLc ratio	≤ 3.0	2.67 (2.41-3.03)	3.25 (2.60-3.60)	1.17 (0.94-1.53)	-0.01 (-0.35-0.31)
	> 3.0	3.56 (3.30-4.16)^**^	4.12 (3.70-4.73)^**^	2.60 (2.16-3.48)^**^	0.79 (0.44-1.39)^**^
Non-HDL cholesterol (mg/dl)	< 130	3.13 (2.70-3.52)	3.75 (3.25-4.46)	1.72 (1.14-2.21)	0.40 (0.05-0.84)
	≥ 130	3.41 (2.92-3.86)^*^	3.87 (3.18-4.43)	2.30 (1.67-3.07)^**^	0.61 (0.08-1.02)
hsCRP (mg/l)	< 1.0	3.11 (2.46-3.40)	3.49 (2.83-4.09)	1.74 (1.05-2.41)	0.22 (-0.34-0.62)
	1-3	3.46 (3.07-3.85)^**^	3.77 (3.37-4.23)^*^	2.29 (1.76-3.09)^**^	0.66 (0.28-1.04)^**^
	≥ 3.0	3.62 (3.07-4.50)^**^	4.24 (3.41-5.12)^**,‡^	2.52 (1.77-3.50)^**^	1.02 (0.43-2.02)^**,‡^
eGFR (ml/min/1.73 m^2^)	≥ 90	3.16 (2.64-3.46)	3.32 (2.90-3.93)	1.87 (1.26-2.57)	0.31 (-0.10-0.75)
	60-89	3.45 (2.92-4.11)^**^	3.96 (3.38-4.63)^**^	2.24 (1.37-3.14)	0.66 (0.16-1.44)^**^
	< 60	3.38 (3.14-3.93)^**^	4.08 (3.89-4.74)^**^	2.18 (1.68-2.77)	0.82 (0.30-1.13)^**^
Proteinuria	Negative	3.11 (2.71-3.39)	3.36 (2.83-3.92)	1.85 (1.34-2.67)	0.31 (-0.01-0.70)
	1+ or more	3.45 (2.93-3.98)^**^	4.00 (3.42-4.66)^**^	2.19 (1.49-3.07)	0.67 (0.09-1.36)^**^

Date expressed as mean ± standard deviation, median (interquartile range), n (%). Mann-Whitney U test. ^*^P < 0.05 and ^**^P < 0.01 with respect to the first category of the biomarker. ^‡^P < 0.05 and ^‡‡^P < 0.01 with respect to the second category of the biomarker.

BMI = body mass index; WC = waist circumference; WHR = waist to height ratio; SBP = systolic blood pressure; DBP = diastolic blood pressure; HbA1_C_ = A1_C_ hemoglobin fraction; TC = total cholesterol; LDLc = low-density lipoprotein cholesterol; HDLc = high-density lipoprotein cholesterol; TGL = triglycerides; hsCRP = ultrasensitive C-reactive protein; eGFR = estimated glomerular filtration rate.

The linear regression analysis revealed that all the scores were positively correlated with BMI, waist circumference, WHR, %BF, glucose, HbA1_C_, TGL, TC/HDLc, LDLc/HDLc ratio, TGL/HDLc ratio, non-HDL cholesterol, hsCRP and degree of proteinuria; all the scores were negatively correlated with HDLc. The siMS score, siMS risk score and MetS Z-score were inversely correlated with the eGFR (Table 3). None of the scores studied showed correlations with LDLc after adjustment for age and sex.

**Table 3.** Multiple linear regression analysis on continuous scores for cardiometabolic risk and biomarkers for cardiometabolic risk, inflammation and glomerular function, adjusted for age and sex

Biomarkers	siMS score		siMS risk score		ICMet		MetS Z-score	
	β (SE)	P	β (SE)	P	β (SE)	P	β (SE)	P
BMI	0.061 (0.014)	< 0.001	0.056 (0.018)	0.002	0.065 (0.021)	0.002	0.079 (0.015)	< 0.001
WC	0.026 (0.006)	< 0.001	0.022 (0.008)	0.003	0.026 (0.009)	0.004	0.037 (0.007)	< 0.001
WHR	4.345 (0.958)	< 0.001	4.236 (1.191)	< 0.001	4.848 (1.435)	0.001	5.714 (1.038)	< 0.001
Body fat %	0.027 (0.010)	0.006	0.024 (0.012)	0.049	0.028 (0.015)	0.049	0.024 (0.011)	0.030
SBP	---	---	0.010 (0.004)	0.009	---	---	---	---
DBP	0.013 (0.006)	0.039	0.022 (0.008)	0.005	0.017 (0.009)	0.049	---	---
Glucose	0.013 (0.001)	< 0.001	0.013 (0.002)	< 0.001	0.011 (0.002)	< 0.001	0.017 (0.001)	< 0.001
HbA1_C_	0.176 (0.025)	< 0.001	0.183 (0.032)	< 0.001	0.158 (0.040)	< 0.001	0.243 (0.025)	< 0.001
TC	---	---	0.003 (0.001)	0.045	0.004 (0.002)	0.016	---	---
LDLc	---	---	---	---	---	---	---	---
HDLc	-0.036 (0.005)	< 0.001	-0.031 (0.007)	< 0.001	-0.052 (0.008)	< 0.001	-0.037 (0.006 )	< 0.001
TGL	0.008 (0.001)	< 0.001	0.009 (0.001)	< 0.001	0.013 (0.001)	< 0.001	0.007 (0.001)	< 0.001
TC/HDLc ratio	0.243 (0.034)	< 0.001	0.254 (0.044)	< 0.001	0.392 (0.049)	< 0.001	0.234 (0.039)	< 0.001
LDLc/HDLc ratio	0.169 (0.043)	< 0.001	0.167 (0.054)	0.002	0.226 (0.064)	0.001	0.149 (0.048)	0.002
TGL/HDLc ratio	0.345 (0.017)	< 0.001	0.366 (0.027)	< 0.001	0.573 (0.012)	< 0.001	0.333 (0.023)	< 0.001
Non-HDL cholesterol	0.004 (0.001)	0.001	0.004 (0.001)	< 0.001	0.006 (0.002)	< 0.001	0.002 (0.001)	0.049
hsCRP	0.041 (0.008)	< 0.001	0.044 (0.010)	0.011	0.037 (0.012)	0.002	0.054 (0.008)	< 0.001
eGFR	-0.011 (0.003)	0.001	-0.013 (0.004)	0.001	---	---	-0.015 (0.004)	< 0.001
Semi-quantified proteinuria	0.724 (0.198)	< 0.001	0.791 (0.243)	0.001	0.674 (0.297)	0.024	0.769 (0.221)	0.001

SE = standard error; BMI = body mass index; WC = waist circumference; WHR = waist to height ratio; SBP = systolic blood pressure; DBP = diastolic blood pressure; HbA1_C_ = A1_C_ hemoglobin fraction; TC = total cholesterol; LDLc = low-density lipoprotein cholesterol; HDLc = high-density lipoprotein cholesterol; TGL = triglycerides; hsCRP = ultrasensitive C-reactive protein; eGFR = estimated glomerular filtration rate.

The predictive value of the scores studied for the entire sample and according to sex are shown in Table 4 and Table 5. In the entire sample, MetS Z-score, siMS score and siMS risk score showed the ability to detect or discriminate metabolic syndrome, reduced glycemic control (HbA1_C_ ≥ 5.7%), proinflammatory state (hsCRP ≥ 1 mg/l) and decreased eGFR (< 90 ml/min/1.73 m^2^); ICMet only had significant capacity to discriminate patients with metabolic syndrome and a proinflammatory state. Overall, the AUCs for MetS Z-score were significantly higher than the AUCs for the rest of the scores for discriminating metabolic syndrome, decreased glycemic control and proinflammatory state. Only the AUCs for MetS Z-score and siMS score for metabolic syndrome were similar. For reduced eGFR, the AUC for the siMS risk score was greater but did not differ significantly from the AUCs corresponding to siMS score and MetS Z-score.

**Table 4.** Area under the curve for continuous scores for cardiometabolic risk of detection of metabolic syndrome, reduced glycemic control, proinflammatory state and decreased estimated glomerular filtration rate, for the entire group

Continuous scores	AUC (95% CI)	SE	P^a^	P^b^
Metabolic syndrome				
siMS score	0.930 (0.881-0.963)	0.0179	< 0.0001	0.2028
siMS risk score	0.763 (0.715-0.841)	0.0341	< 0.0001	< 0.0001
ICMet	0.871 (0.812-0.917)	0.0265	< 0.0001	0.0012
MetS Z-score	0.942 (0.896-0.971)*	0.0172	< 0.0001	----
Reduced glycemic control: HbA1_C_ ≥ 5.7%				
siMS score	0.654 (0.579-0.724)	0.041	0.0002	< 0.0001
siMS risk score	0.607 (0.531-0.680)	0.043	0.0121	0.0015
ICMet	0.574 (0.497-0.648)	0.044	0.0902	< 0.0001
MetS Z-score	0.723 (0.651-0.788)*	0.038	< 0.0001	----
Proinflammatory state: hsCRP ≥ 1 mg/l				
siMS score	0.779 (0.667-0.802)	0.037	< 0.0001	0.0036
siMS risk score	0.668 (0.593-0.737)	0.041	< 0.0001	0.0007
ICMet	0.677 (0.603-0.746)	0.042	< 0.0001	0.0002
MetS Z-score	0.780 (0.712-0.839)*	0.034	< 0.0001	----
Decreased glomerular function: eGFR < 90 ml/min/1.73 m^2^				
siMS score	0.658 (0.581-0.779)	0.042	0.0002	0.6592
siMS risk score	0.676 (0.600-0.745)*	0.043	0.0001	----
ICMet	0.588 (0.510-0.663)	0.046	0.0562	0.0521
MetS Z-score	0.662 (0.586-0.733)	0.042	0.0001	0.7461

^a^Significance level for the null hypothesis AUC = 0.05; ^b^significance level for comparison of AUCs with respect to the AUC that was greatest (^*^). AUC = area under the curve; CI = confidence interval; SE = standard error; HbA1_C_ = A1_C_ hemoglobin fraction; hsCRP = ultrasensitive C-reactive protein; eGFR = estimated glomerular filtration rate.

**Table 5.** Area under the curve for continuous scores for cardiometabolic risk of detection of metabolic syndrome, reduced metabolic control, proinflammatory state and decreased estimated glomerular filtration rate, according to sex

Condition to be detected	siMS score	siMS risk score	ICMet	MetS Z-score
Women				
Metabolic syndrome	0.926 (0.853-0.970)***	0.828 (0.736-0.898)***	0.873 (0.788-0.933)***	0.948 (0.882-0.983)***
HbA1_C_ ≥ 5.7%	0.577 (0.471-0.679)	0.579 (0.473-0.680)	0.515 (0.409-0.619)	0.649 (0.544-0.745)**
hsCRP ≥ 1 mg/l	0.706 (0.603-0.796)**	0.687 (0.52-0.779)**	0.642 (0.536-0.779)*	0.775 (0.677-0.855)**
eGFR < 90 ml/min/1.73 m^2^	0.597 (0.489-0.699)	0.680 (0.489-0.699)**	0.525 (0.417-0.631)	0.594 (0.486-0.696)
Men				
Metabolic syndrome	0.935 (0.859-0.978)***	0.733 (0.624-0.825) ***	0.884 (0.794-0.944)***	0.947 (0.874-0.984)***
HbA1_C_ ≥ 5.7%	0.753 (0.646-0.842)***	0.655 (0.542-0.757)**	0.648 (0.535-0.751)**	0.814 (0.712-0.891)***
hsCRP ≥ 1 mg/l	0.769 (0.663-0.855)***	0.642 (0.528-0.745)*	0.721 (0.611-0.815)***	0.789 (0.685-0.872)***
eGFR < 90 ml/min/1.73 m^2^	0.713 (0.600-0.809)***	0.667 (0.552-0.769)**	0.656 (0.540-0.759)**	0.732 (0.620-0.825)***

Data expressed as AUC (95% confidence interval).

^*^P < 0.05 for the null hypothesis AUC = 0.05. ^**^P < 0.01 for the null hypothesis AUC = 0.05. ^***^P < 0.0001 for the null hypothesis AUC = 0.05.

AUC = area under the curve; HbA1_C_ = A1_C_ hemoglobin fraction; hsCRP = ultrasensitive C-reactive protein; eGFR = estimated glomerular filtration rate.

Among women, all the scores assessed significantly discriminated metabolic syndrome and proinflammatory state. Only MetS Z-score had the capacity to detect reduced glycemic control, while siMS risk score showed the ability to discriminate reduced eGFR. Among men, all the scores had predictive value for detecting the conditions studied.

## DISCUSSION

The main purpose of this study was to examine the validity of four continuous scores that had been proposed for quantification of cardiometabolic risk. Overall, the four scores showed significant associations with most of the anthropometric and biochemical biomarkers that were measured. The scores studied showed predictive value for metabolic syndrome, reduced glycemic control, proinflammatory state and reduced estimated glomerular function, with small differences in performance especially regarding the levels of glycemic control and glomerular filtration. Metabolic syndrome was the condition for which all the scores had the greatest ability to discriminate, as expected, since all the scores were calculated using the same individual components of metabolic syndrome. In addition, all the scores increased progressively and significantly as the number of individual metabolic syndrome components increased, thus showing a continuous and gradual relationship between the scores tested and the accumulation of cardiometabolic risk factors. This behavior is desired for continuous measurements.

Out of the four scores studied, the MetS Z-score provided the most information for comparisons and for discussing its utility. In our study, after adjusting for sex and age, this score correlated with BMI, waist circumference, WHR, %BF, glucose, HbA1_C_, HDLc, TGL, TC/HDLc ratio, LDLc/HDLc ratio, TGL/HDLc ratio, non-HDL cholesterol, hsCRP, eGFR and degree of proteinuria. In both men and women, it could detect metabolic syndrome, reduced glycemic control and proinflammatory state. These results are consistent with the associations found by Gurka et al.^24^ between MetS Z-score and risk factors, along with its ability to predict the progression of coronary heart disease and diabetes.^28^^,^^29^^,^^30^^,^^31^

The MetS Z-score can also be highlighted as having the highest AUC for detecting three of the four conditions studied. In particular, it was the only score able to discriminate HbA1_C_ values ≥ 5.7% among women. The latter probably reflects the load factor that was obtained from glucose in constructing the equations for MetS Z-score, which was > 0.4 in women.^24^

DeBoer et al.^32^ corelated elevation of the MetS Z-score with declining eGFR, higher prevalence of microalbuminuria and higher incidence of chronic kidney disease in African-American women. In our entire sample, MetS Z-score correlated negatively with eGFR and positively with the degree of proteinuria. Nevertheless, it was only able to discriminate eGFR < 90 ml/min/1.73 m^2^ in males. This divergence may have been due to racial differences that affect susceptibility to deterioration of glomerular function and the distribution of the components of metabolic syndrome.

We assessed ICMet because it includes a few simple determinations that provide information on the metabolism of triglyceride-rich lipoproteins, insulin resistance and glycemic control. Wakabayashi and Daimon^25^ found a positive association between ICMet and HbA1_C_ and showed that ICMet had significant predictive value for detecting diabetes and hyperglycemia (HbA1_C_ ≥ 5.7%) in Japanese women and men. Associations between ICMet and smoking habits,^33^ progression of atheromatous plaque in patients with peripheral arterial disease^34^ and the risk of hypertension^35^ have also been reported.

In the present study, higher ICMet was observed in individuals with HbA1_C_ ≥ 5.7%, metabolic syndrome or diabetics. Likewise, ICMet correlated with most of the biomarkers that were measured, after adjustment for sex and age (except for systolic pressure, LDLc and degree of proteinuria). However, this measurement only had the capacity to detect metabolic syndrome and proinflammatory status in the entire sample and only showed predictive value for decreased glycemic control and reduced eGFR among men. ICMet also did not vary significantly among smokers or hypertensive patients. These observations place some doubt on the applicability of ICMet as a continuous measurement of cardiometabolic risk in our population. This needs to be elucidated through other studies.

The siMS score and siMS risk score were only proposed in the year 2016 and there is no further information about their performance. Their authors^26^ reported that both scores correlated strongly with other indexes and that there was a medium-high grade correlation between siMS risk score and the Framingham score in a group of adult patients in Belgrade, Serbia.

In our entire sample, siMS score and siMS risk score correlated significantly with the anthropometric and biochemical biomarkers that were measured and had the capacity to detect metabolic syndrome, decreased glycemic control, proinflammatory state and reduced eGFR. However, the siMS risk score showed better performance. It was the only score that showed significant variation among smokers. In addition, the siMS risk score showed the ability to detect all the above conditions among men and to discriminate reduced glomerular filtration in women. The siMS score depends only on metabolic syndrome components. The siMS risk score is time-dependent because it incorporates age and heritability, so it is likely that its performance is better because it takes into consideration the progressive evolution of cardiometabolic risk and the genetic component involved in cardiometabolic diseases. The findings potentially support use of the siMS risk score as a continuous measurement of cardiometabolic risk, but it will be important to determine its ability to predict cardiovascular events or development of diabetes, through prospective studies.

Our attention was drawn to the fact that none of the four scores tested substantially differed with regard to LDLc and hypertension and only one (the siMS risk score) shown any significant correlation with systolic blood pressure. MetS Z-score did not have any correlation with either of the two components of blood pressure, and this finding can be partially explained by the low load factor (< 0.4) that was ascertained in relation to systolic pressure in the principal component analysis from which the equations for MetS Z-score originated.^24^ In principle, these observations preclude implementation of the scores tested here, among patients with hypertension or with hypercholesterolemia alone. However, it is important to note that most of the participants in this study were undergoing treatment with antihypertensive agents or other drugs. This may have affected the results observed and therefore other investigations may be required.

The present work has some limitations. In the first place, the results found need to be confirmed through using a more extensive sample. The present results were observations derived from a group of individuals who were enrolled in a control program for chronic non-communicable diseases and their risk factors, and therefore the findings cannot be extrapolated to the general population. Secondly, the cross-sectional nature of the study and the lack of follow-up among the patients, to observe the incidence of cardiovascular events or diabetes, precluded calculation of cutoff points for stratifying the cardiometabolic risk according to the scores assessed. This latter point seems to be contradictory, given the limitations of dichotomous classifications, but it remains useful within clinical practice, for identifying patients who require strong intervention. It also forms a tangible goal for patients and their physicians, thereby functioning as a quantitative measurement of progress or deterioration.

## CONCLUSION

In a sample of Venezuelan adults, all the scores studied varied according to different anthropometric and biochemical biomarkers for cardiometabolic risk. They showed predictive value for metabolic syndrome and proinflammatory status. Three scores showed a predictive capacity regarding reduced glycemic control and decreased renal glomerular function. Because this study found certain differences in the performance of the scores studied, especially with regard to sex, selection of one or another will depend on the aim and the scope pursued. The aim in follow-up studies will be to confirm the present findings and their usefulness for prevention and intervention protocols relating to cardiometabolic diseases.
